# Management of non-ovarian cancer malignant ascites through indwelling catheter drainage

**DOI:** 10.1186/s12904-016-0116-5

**Published:** 2016-04-21

**Authors:** Xiaoli Gu, Yuanyuan Zhang, Menglei Cheng, Minghui Liu, Zhe Zhang, Wenwu Cheng

**Affiliations:** Department of Integrated Therapy, Fudan University Shanghai Cancer Center, Shanghai, 200032 China; Department of Oncology, Shanghai Medical College, Fudan University, Shanghai, 200032 China; #270, DongAn Road, Shanghai, 200032 People’s Republic of China

**Keywords:** Malignant ascites, Paracentesis, Indwelling catheter, Drainage, Symptom management, Palliative care

## Abstract

**Backgrounds:**

Intra-abdominal placement of the Central Venous Catheter (CVC) was conducted to manage the ascites-related symptoms of non-ovarian cancer patients. The aim of this study is to document the efficacy of symptom relief and conduct survival analysis of non-ovarian cancer patients with malignant ascites who received paracentesis and indwelling catheter drainage.

**Methods:**

Seventy eight patients received paracentesis and drainage. All patients who met the inclusion criteria were included in this study. The overall survival (OS) was defined as the interval between initial diagnosis and death. Since-paracentesis survival (SP-Survival) was defined as the interval between initial paracentesis and death.

**Results:**

Hepatic cancer was the most frequent original cancer in this study. Peritoneal catheters remained in situ for a median of 13 days. No immediate complications, such as perforation of a viscus or excessive bleeding, were encountered during placement. All ascites-related symptoms improved after drainage compared with the baseline. There was a statistically significant improvement in the mean score for abdominal swelling (*p* < 0.001), anorexia (*p* = 0.023) and constipation (*p* = 0.045). Cancer type was shown to be an independent prognostic factor for overall survival length (*p* = 0.001). Serum albumin was an independent prognostic factor for SP-survival (*p* = 0.02).

**Conclusions:**

Paracentesis and indwelling catheter drainage through CVC set is a useful method for improving painful symptom. Further research is needed to validate the findings.

## Background

Malignant ascites (MA) is defined as abnormal accumulation of fluid within the peritoneal cavity caused by the intraperitoneal spread of original cancer [1]. It accounts for 10 % of all cases of ascites. 6 % of all admitted palliative care patients receive treatment for MA [2].

MA is usually a sign of end-stage illness that is accomplanied by abdominal pain, discomfort, anorexia, nausea, and dyspnea. These ascites-related symptoms seriously affect patients’ quality of life (QOL) [3, 4]. Except for ovarian cancer, MA confers a poor prognosis, with a median survival time of 5.7 months [5]. For gastrointestinal cancer patients with MA, the median overall survival (OS) is less than 3 months [6]. Therefore the therapy for advanced non-ovarian cancer patients with MA is generally aimed at managing ascites-related symptoms [7], alleviating patients’ sufferings, and improving their QOL [8–10].

Limited treatment options exist for advanced cancer patients with MA. These patients not only have poor general status but also respond poorly to dietary sodium restriction and diuretics [11, 12]. Effective palliation of MA remains a challenge [13]. Paracentesis and drainage are widely performed for patients with MA whose considered therapeutic goal is palliation [14, 15]. However, the effect of paracentesis is short-lived and has to be repeated every 10 days on average [16]. Continuous peritoneal drainage through an indwelling catheter has been reported to be hugely beneficial to symptom management, avoiding the hazards and disadvantages-direct and indirect complications-of multiple repeated procedures [17, 18].

Although placement of indwelling catheters for symptom relief has been a recommended therapy for end-stage patients with MA, there is scant data in the existing literature that documents the efficacy of symptom relief and includes follow-up survival analysis [19, 20]. We conducted a retrospective review of all non-ovarian cancer patients who received indwelling catheters for the management of symptomatic MA over a 3-year period. Considering the different biomedical behaviors and therapeutic responses of patients with ovarian cancer and MA [21–23], ovarian cancer patients were excluded from this study. The aim of this study is to document the efficacy of symptom relief and to conduct a survival analysis of non-ovarian cancer patients with MA who received paracentesis and indwelling catheter drainage.

## Methods

### Patients

From March 2008 to March 2011, 345 cancer patients were admitted to the Integrated Therapy Department (also known as Palliative Care Unit, PCU) of Fudan University Shanghai Cancer Center (FUSCC). 145 patients were diagnosed with MA. The inclusion criteria consisted of the following: 1. Patients’ age was not less than 18 years at the time of enrollment. 2. Patients had histologic or cytologic proof of original malignancy and/or MA. 3. Patients had no chance to receive anti-cancer treatments for malignancy, including systematic chemotherapy, radiotherapy and etc. 4. Patients received intra-peritoneal catheterization and drainage during the interval to relieve symptom sufferings. 5. A coagulation screen, comprised of platelet count, prothrombin time (PT) and activated partial thromboplastin time (aPTT), was performed prior to the procedure to exclude contraindications. Of the 145 patients diagnosed with MA, 78 patients met the inclusion criteria and were included in this study. The international Classification of Disease-9 (ICD-9) coding system was used to code the medical records of all patients.

Medical records were reviewed by three attending physicians through the Union Medical System (UMS) of FUSCC to gather general characteristics, cancer-related information, symptoms, and ascites-related records. The UMS is the electronic medical records system containing all the medical information of patients in the FUSCC, including demographic and clinical data.

### Catheter insertion procedure

**Fig. 1:**
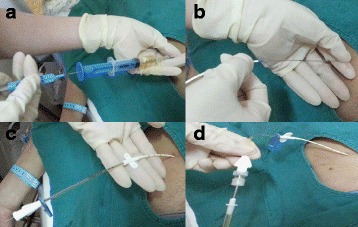
The catheter insertion procedure. **a** A spring wire guide was introduced into peritoneal cavity through the introducer needle. **b** The indwelling catheter was put into the peritoneal cavity following the spring wire guide after the abdominal wall was dilated. **c** The spring wire guide was extracted gradually with the catheter pushing forward into the peritoneal cavity. **d** The catheter outlet attached to a Heparin Cap and a governor

**Fig. 2:**
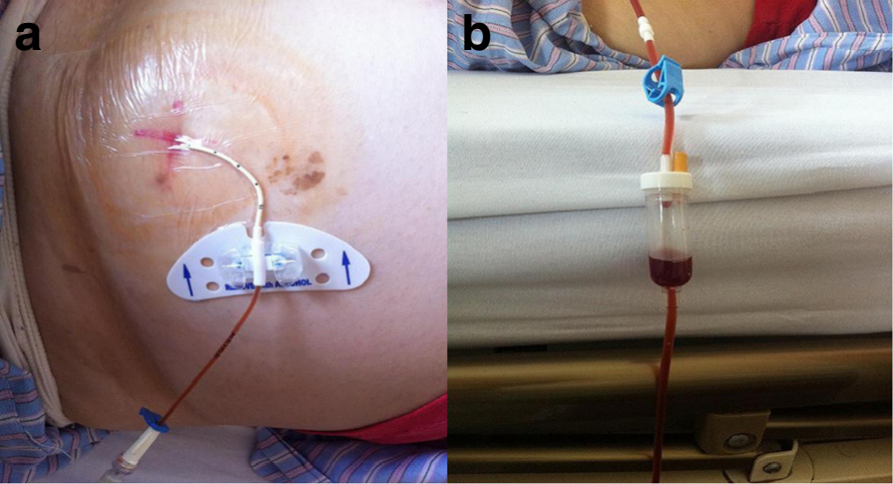
The indwelling catheter and drainage governor. **a** The catheter was fixed to abdominal wall by Statlock; **b** The governor for intravenous infusion was used to help control the rate of fluid removal

Informed consent for paracentesis and drainage was obtained before the procedure was performed. The procedure was carried out by patients’ attending physicians with more than 5 years’ clinical experience bedside with the patient supine. Patients were asked to urinate before the procedure. The CVC set used (Arrow Raulerson Syring, USA, REF ES-04306), which contains multiple side-holes in addition to an end-hole, was initially developed for deep vein indwelling and allowed for improved drainage and decreased risk of catheter blockage. The puncture site was located by ultrasonographic imaging prior to the procedure. 5–10 ml 2 % lidocaine was used for local anesthesia. An introducer needle was inserted into the peritoneal cavity, and a spring wire guide was placed in the peritoneal cavity through the introducer needle. The needle was then extracted. The indwelling catheter was placed in the peritoneal cavity following the spring wire guide after the abdominal wall was dilated. The spring wire guide was then extracted gradually with the catheter pushing forward into the peritoneal cavity. The catheter outlet was attached to a Heparin Cap (Heatlth Care Ltd) and a governor (part of the Infusion Sets with Precision Filters for Single Use, Wuhan W.E.O. Science&Technique Development Co, Ltd), then connected to a drainage bag (Fig. 1). Finally, the catheter was fixed to the abdominal wall by Statlock (Catheter Stabilization Device, Bard Access Systems, Inc). The governor for intravenous infusion was used to help control the rate of fluid removal and prevent sudden rapid fluid loss (Fig. 2). Especially for patients with tense ascites, the high tension in the peritoneal cavity can result in rapid drainage. We controlled the rate at about 300 ml per hour (85drops/min) for patients. 1000–1500 ml ascites were aspirated on the first day. The fluid of the first day was used for biochemical tests and fluid cytology. Drainage was continued or stopped according to the patients’ suffering and specific situation. Drainage was discontinued if disease progression or intolerable toxicities were confirmed or patients refused further treatments. In order to avoid hypovolemia and paracentesis-induced circulatory dysfunction (PICD), infusion of albumin and synthetic plasma expanders was used for all patients during the drainage interval. The diuretics were used and adjusted according to patients’ weight loss and electrolyte levels. The volume of extracted ascites and immediate and late complications including hypotension, haemorrhage, tube blockage, dislodgment and sepsis, were recorded in chart.

### Evaluation

The symptoms related with ascites were assessed before paracentesis and then at the end-point of catheter removal. The most frequent ascites-related symptoms included fatigue, abdominal swelling, anorexia, abdominal pain, constipation, dyspnea, nausea and vomiting, insomnia, early satiety, and dizziness. The severity of the symptoms was assessed on a scale from 0 to 10, with zero denoting no symptom sufferings and ten representing symptoms as severe as the patients could imagine. The mean score of ascites-related symptoms was calculated and pre- and post-treatment scores were compared. Patient data was retrospectively reviewed through the charts.

Patient follow up took place 1 week after discharge and monthly for 6 months, then once every 3 months until death. The data were obtained from phone call records of the follow-up information of the Department. The overall survival (OS) was defined as the interval post-disease diagnosis till death (months). Since-paracentesis survival (SP-survival) was defined as the interval post-initial paracentesis till death (days).

### Statistical analysis

Continuous variables were compared using the *t*-test for samples, while frequencies and rates were compared using the chi-square test. Symptom scores were presented as mean ± standard error. Pre-and post-treatment symptom scores were compared using a *t*-test. Values of *p* < 0.05 were considered to be statistically significant. The relationship between each variable and survival time was analyzed by the Kaplan-Meier method and Cox proportional hazards models. Statistical analysis was performed using Statistical Package for the Social Sciences (SPSS) software Version 16.0 for Windows (SPSS Inc., Chicago, USA).

## Results

### Patients

During the study period, 178 patients were diagnosed with MA. Eighty two non-ovarian cancer patients accepted paracentesis and indwelling catheter drainage. Seventy eight patients were eligible for this study, with 43 male and 35 females. The mean age of those included was 58 years old (95 % CI: 55–62). The most frequent original cancers causing ascites in this study were hepatic (15, 19.2 %), pancreatic (12, 15.4 %) and colorectal cancer (12, 15.4 %). 59 (75.6 %) patients had visceral metastasis. The liver (59.0 %) was the most prevalent metastatic site, and 38 patients (48.7 %) had more than three sites of metastasis. 33 (42.3 %) patients were also diagnosed with pleural effusion. Thirty four patients had previously received chemotherapy, with a median of three previous courses of chemotherapy. The most frequent KPS was 40, indicating that patients were mainly bed bound, needing assistance with care.

Insertion of the catheter was technically successful in all 78 cases. 16 patients had two catheters inserted in separated locations. The peritoneal catheters remained in situ for a median of 13 days (range 5–18 days). The mean depth of the ascites before drainage scanned by B-type ultrasound was 93.6 mm (range: 35–143). The medial total serum albumin at admission was 30.1 g/L (range: 21–36). The median volumeof drainages per patient was 5750 ml and the mean volume was 8538 ml (range: 750–53300 ml). Diuretics were used in 70 patients concomitant with the paracentesis. Among these patients, 35 patients used spironolactone alone, ten used furosemide alone, and 25 patients used both.

No immediate complication such as perforation of a viscus or excessive bleeding was encountered during the placement procedure. Seven patients had continuous leakage of peritoneal fluid from the needle site during the drainage. The general characteristics and ascites-related characteristic are shown in Table 1.

**Table 1:** Seventy-eight patients’ general and ascites related characteristics (*N* = 78)

General characteristics	N.	%	Ascites related	N	%
Age			Serum albumin(g/L)		
<=50	21	26.9	<=30	32	41.0
50–70	43	55.1	>30	46	59.0
> = 70	14	17.9	Color		
Gender			Yellow	15	19.2
Male	35	44.9	Brown	43	55.1
Female	43	55.1	Red	20	25.6
Malignancy type			Gravity		
GI group^a^	48	61.5	>1.012	23	29.5
Others^b^	16	20.5	<=1.012	55	70.5
Unknown	14	17.9	Rivalta test		
Metastasis			-	22	28.2
Liver	46	59.0	+	47	60.2
Lung	16	20.5	(++ − +++)	9	11.5
Bone	12	15.4	Ascites LDH		
Treatment History			>200	45	57.7
Surgery	33	42.3	<=200	33	42.3
Chemotherapy	35	44.9	Removed ascites volume		
Radiotherapy	35	44.9	<=3000 ml	16	20.5
Others	22	28.2	3000–5000 ml	25	32.1
Diuretics			> = 5000 ml	37	47.4
Aldosterone antagonist only	35	44.9			
Frusemide only	10	12.8			
Combination of both	25	32.1			

*Rivalta Test* The Rivalta test was used in order to differentiate a transudate from an exudate. A positive the test meant high protein concentration of ascite fluid and possibly a relationship with Spontaneous bacterial peritonitis (SBP)

*Ascites LDH* Ascites lactate dehydrogenase was used to differentiate malignant transudate from benign transudate

^a^GI group: The gastrointestinal group included hepatic cancer, pancreatic cancer, colorectal cancer and etc

^b^Other primary tumors included lung, breast, renal, etc. Unknown is for patients that had malignant proof but not primary proof

### Symptom improvement after drainage

The most common symptom before paracentesis was fatigue, reported in 73 patients (93.6 %). The second most common symptom was abdominal swelling in 72 patients (92.3 %), then anorexia in 59 (75.6 %), and abdominal pain in 36 (46.2 %) patients. For 62 patients, more than 3 symptoms were reported. All the ascites-related symptoms were alleviated after drainage compared with the baseline. There was a statistically significant alleviation in the mean score for abdominal swelling (*p* < 0.001), anorexia (*p* = 0.023) and constipation (*p* = 0.045). Details are shown in Table 2.

**Table 2:** The most frequent ascites related symptoms and change post- paracentesis

Symptoms	N	%	Medina score before drainage	Mean score before drainage	Mean score After drainage	*p* Value
Fatigue	73	93.6	6	6.7	6.4	>0.05
Abdominal swelling	72	92.3	7	5.3	2.4	<0.001*
Anorexia	59	75.6	7	6.2	4.7	=0.023*
Abdominal pain	36	46.2	5	4.5	2.1	>0.05
Constipation	29	37.1	6	5.6	4.5	0.045*
Dyspnea	26	33.3	4	3.2	2.5	>0.05
Nausea &vomiting	18	23.1	5	4.6	3.7	>0.05
Insomnia	30	38.5	4	4.2	2.5	>0.05
Early satiety	10	12.8	4	3.8	3.6	>0.05
dizzy	4	5.1	4	4.5	3.4	>0.05

The pre-paracentesis and post-paracentesis scores of the symptoms assessment were compared using a *t* test of the mean scores

* *p* < 0.05

### Post-drainage survival outcomes

**Fig. 3:**
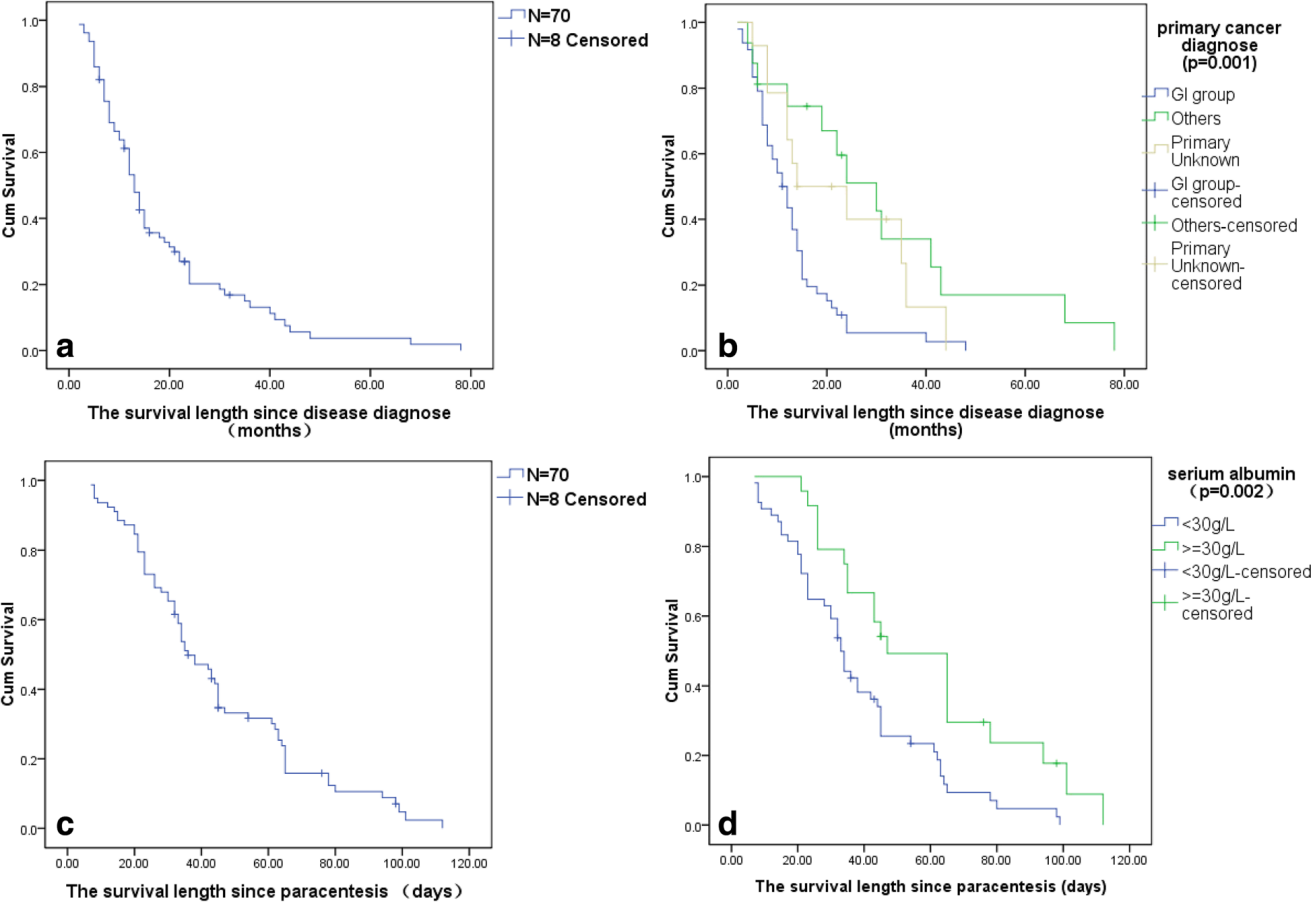
**a** The median overall survival (survival since diagnosis) was 13 months (95 % CI: 11.2–14.8). **b** patients with ascites of GI origin group had the worst overall survival (*p* = 0.001). **c** The median survival from paracentesis and indwelling catheter drainage (SP-Survival) was 36 days (95 % CI: 29.9–43.0). **d** Serum albumin concentration significantly affected survival with a normal serum albumin of greater than 30 g/L being associated with an improved SP-Survival (*p* = 0.02)

Follow-up was conducted until death or at the time of the study (December, 2013). Fourteen patients received indwelling catheter drainage twice. Seventy patients died within the follow-up period and 8 patients were still alive at the time of survival analysis. The median OS was 13 months (95 % CI: 11.2–14.8). Patients with ascites of gastrointestinal origin group had the worst OS (*p* = 0.001) (Fig. 3a, b). The median SP-Survival was 36 days (95 % CI: 29.9–43.0). Serum albumin concentration significantly affected survival with a serum albumin of greater than 30 g/L being associated with an improved SP-Survival (*p* = 0.02) (Fig. 3c, d). Multivariate Cox regression showed cancer type to be an independent prognostic factor for OS and serum albumin to be an independent prognostic factor for SP-Survival. Details are shown in Table 3.

**Table 3:** Multivariate analysis of overall survival and SP-Survival by Cox proportional hazards models

Variables	Overall Survival		SP Survival	
	*p*	Hazard Ratio(95 % CI)	*p*	Hazard Ratio(95 % CI)
Age	0.681		0.656	
<=50	Ref		Ref	Ref
50–70	0.575	1.211 (0.621–2.361)	0.360	1.357(0.707–2.604)
> = 70	0.744	0.858 (0.344–2.140)	0.617	1.246(0.526–2.950)
Gender(male/female)	0.323	1.339 (0.750–2.392)	0.667	0.877(0.484–1.591)
Primary cancer	0.026*		0.315	
GI group	Ref	Ref	Ref	Ref
Others	0.03	0.418 (0.190–0.920)	0.561	0.805(0.388–1.672)
Unknown	0.033	0.423 (0.192–0.932)	0.134	0.566(0.268–1.192)
Ascites related				
Color	0.566		0.693	
Yellow	Ref	Ref	Ref	Ref
Brown	0.291	1.571 (0.680–3.630)	0.543	0.779(0.348–1.742)
Red	0.656	1.167 (0.591–2.304)	0.830	1.074(0.561–2.054)
LDH (>200/<=200)	0.225	0.798 (0.442–2.350)	0.065	0.520(0.275–0.984)
Rivalta Test	0.077		0.745	
-	Ref	Ref	Ref	Ref
+	0.067	0.258 (0.096–0.695)	0.453	1.418(0.570–3.530)
++ − +++	0.088	0.357 (0.141–0.902)	0.507	1.321(0.581–3.003)
Gravity (>1.012/<=1.012)	0.260	0.706 (0.385–1.294)	0.159	0.649(0.356–1.185)
Volume removed	0.904		0.357	
<=3000 ml	Ref	Ref	Ref	Ref
3000–5000 ml	0.701	1.161 (0.542–2.484)	0.873	0.939(0.433–2.035)
> = 5000 ml	0.961	0.985 (0.537–1.806)	0.249	1.494(0.755–2.958)
Serum albumin (<30 g/L/> = 30 g/L)	0.394	0.759 (0.402–1.432)	<0.001*	0.258(0.128–0.517)

Primary cancer diagnosis had a significant relationship with the overall survival (*p* = 0.026). The serum albumin level had a significant relationship with SP-Survival (*p* < 0.001)

*p < 0.05

## Discussion

The mechanism of production of MA is a complex, multi-factorial process [24]. No validated guidelines for preventing or reducing the production reaccumulation of MA currently exist [25]. Though indwelling peritoneal drainage catheters were considered to be the first choice for patients with MA who received palliative care [26], there were no international standards or guidelines for paracentesis and drainage, or specially designed catheters for continuous peritoneal drainage [27]. Previous studies reported the successful use of many different catheters for refractory ascites [28, 29]. At first, the CVC set was not used for abdominal paracentesis. Previous studies testified its safety, convenience and fewer complications for intraperitoneal chemotherapy in advanced gastric cancer [30]. In our department, the CVC set for Seldinger technique was used. This technique has been successfully applied for 8 years in our department. This retrospective study demonstrated that subcutaneous peritoneal indwelling catheters through CVC were safe, feasible, and effective for terminally ill cancer patients with MA.

The CVC used as the indwelling catheter offered lots of potential advantages. The technique is a simple method that can be performed by both trained physicians and internal medicine residents. Whereas some other methods are more invasive and require an operating room, this procedure can be performed at the bedside. [31]. Less immediate complications, such as perforation of a viscus or excessive bleeding, were encountered during the catheter insertion, drainage and catheter extraction. The short placement of the catheter obviously decreased the catheter-induced peritonitis that is normally caused by the catheter remaining in the patient for long periods of time [32, 33]. Further studies to investigate the use of CVC on patient outcomes should be conducted.

It had been suggested that reducing the flow rate of ascites extraction may help to prevent PICD [34]. Use of the governor allowed for the drainage rate to be controlled according to patients' condition and blood pressure. The drainage volume can be adjusted from a few litres to a maximum of over 20 L. In order to further guarantee the safety of this technique, intravenous infusions were administered to avoid negative effects like hypovolemia. Albumin infusion was used routely for these patients. The albumin infusion increased invascular oncotic pressure and caused mobilization of fluids from the interstitium into the intravascular space. Some studies showed that the recurrence of ascites decreased for patients with good diuretic response when combined with albumin. Previous studies reported that albumin infusion reduced the incidence of post-paracentesis circulatory dysfunction by 15–20 % [35]. However, controversy still existed for albumin infusion for refractory ascites [36]. One study demonstrated that only 11 % of the oncologists in a previous survey used human albumin when patients received paracentesis and drainage [37].

Previous study have reported that apart from ovarian cancer, gastrointestinal cancer was the most common tumor of origin [38], with up to 15 % of all patients with gastrointestinal cancers developing ascites at some stage of their disease [39]. In our study, hepatic cancer had the highest proportion. This may be due to the high incidence of primary hepatocellular carcinoma in the population of mainland China. The percentage of unknown original tumors was 17.9 %, similar with the 20 % of all patients with MA in previous studies [40].

Non-resolving or recurrent MA was always considered to be associated with poor survival [41]. However, the prognostic factors associated with MA had been poorly studied. One study that focused on pancreatic cancer testified that ascites was a harbinger of the final stage of pancreatic cancer and the median survival after ascites occurred was usually between 2 to 4 months [42]. Another retrospective review of 76 patients with MA demonstrated that the median survival following ascites diagnosis was 2.25 months. In our research, the survival since ascites occurred was not analyzed because the retrospective method did not allow for precise determination of the date of ascites diagnosis. Our research focused on survival since original cancer diagnosis and survival since paracentesis, which also reflected that patients with MA were associated with minimal remaining life expectancy. The unknown original group had a better survival than GI origin group in this study. One potential explanation might be that we could not exclude some ovarian cancer patients from the unknown original group, as some ovarian cancer cannot be diagnosed despite together with MA. These patients may resulted in the unknown original group had a better survival in this research.

Although the usage of albumin is still controversial, previous studies testified that the a low level of serum albumin was an independent prognostic factor adversely affecting survival for non-ovarian cancer patients with MA [43, 44]. In this study, patients in the low level serum albumin group had poorer prognoses, and the median survival since paracentesis for this group was only 35 days. The low serum albumin concentration was always accompanied by liver dysfunction and poor protein concentrations. Prospective research focused on the effectiveness of albumin infusion and relationships with the serum albumin concentration of these patients needs to be conducted.

Symptoms related to refractory ascites usually negatively impacts patients’ quality of life [32]. Although patients’ clinical outcomes cannot be altered, and the estimated survival time is limited, symptoms relief should be the treatment goal for these advanced non-ovarian cancer patients with MA. As the disease advanced and symptoms worsened, the refractory ascites dramatically exacerbated several symptoms of patients with terminal malignancies [10]. Paracentesis was testified to relieve these symptoms in 90 % of patients [45]. In our study, all the patients experienced benefits in the form of at least one symptom relieved. The abdominal swelling, anorexia, and constipation were relieved significantly. In fact, symptoms were implicative of each other. For instance, untreated abdominal swelling could lead to nausea, pain, constipation, dyspnea and vice versa. But the symptom alleviation was not satisfactory. Some symptoms’ burden had little change despite the effective removal of large volumes of abdominal fluid. The multifactorial causes of the symptoms may explain this observation.

Limitations of this study include selection bias due to the retrospective nature of the study and a small sample size. Further well powered randomized controlled studies should be conducted to analyze the influence of the use of diuretics, which was used based on each patient’s specific circumstances. Further prospective and randomized studies should be conducted to examine the use of diuretics with paracentesis in the management of MA and to clarify the effect of diuretic usage in MA patients with indwelling catheters for drainage. Prospective studies focused on the combination of efficacy, symptoms improvement assessment, survival length, and patient satisfaction will help to describe the role of indwelling catheters in patients with advanced cancer with MA.

## Conclusion

Our study retrospectively detailed the care of 78 advanced non-ovarian cancer patients with MA. Paracentesis and indwelling catheter drainage through CVC set was a useful method to improve symptom sufferings. Future research is needed to validate these findings.

### Availability of data and marerials

Annonymised data is held at Department of Integrated Therapy, FUSCC. It is not possible to share the data due to regulations states in the ethical approval.

### Consent to publish

This study does not contain any individual person data.

### Ethics and consent to participate

This study protocol was approved by the ethical review board of FUSCC.

## Abbreviation

aPTT: activated partial thromboplastin time
Ascites LDH: Ascites lactate dehydrogenase
CVC: Central Venous Catheter
FUSCC: Fudan University Shanghai Cancer Center
ICD-9: International Classification of Disease-9
MA: malignant ascites
OS: overall survival
PICD: paracentesis-induced circulatory dysfunction
PT: prothrombin time
QOL: quality of life
SPSS: Statistical Package for the Social Sciences
SP-survival: since-paracentesis survival
UMS: Union Medical System
